# Internal construct validity of the Shirom-Melamed Burnout Questionnaire (SMBQ)

**DOI:** 10.1186/1471-2458-12-1

**Published:** 2012-01-03

**Authors:** Åsa Lundgren-Nilsson, Ingibjörg H Jonsdottir, Julie Pallant, Gunnar Ahlborg

**Affiliations:** 1 Institute of Neuroscience and Physiology, Department of clinical neuroscience and rehabilitation, The Sahlgrenska Academy, University of Gothenburg, Per Dubbsgatan 14, plan 3, 413 45 Göteborg, Sweden; 2 Institute of Stress Medicine (ISM), Gothenburg, Sweden; 3 Rural Health Academic Centre, University of Melbourne, Melbourne, VIC, Australia; 4 Department of Public Health and Community Medicine at the Sahlgrenska Academy, University of Gothenburg, Sweden

**Keywords:** Exhaustion disorder, Rasch, SMBQ, Stress, Psychometrics, Work

## Abstract

**Background:**

Burnout is a mental condition defined as a result of continuous and long-term stress exposure, particularly related to psychosocial factors at work. This paper seeks to examine the psychometric properties of the Shirom-Melamed Burnout Questionnaire (SMBQ) for validation of use in a clinical setting.

**Methods:**

Data from both a clinical (319) and general population (319) samples of health care and social insurance workers were included in the study. Data were analysed using both classical and modern test theory approaches, including Confirmatory Factor Analysis (CFA) and Rasch analysis.

**Results:**

Of the 638 people recruited into the study 416 (65%) persons were working full or part time. Data from the SMBQ failed a CFA, and initially failed to satisfy Rasch model expectations. After the removal of 4 of the original items measuring tension, and accommodating local dependency in the data, model expectations were met. As such, the total score from the revised scale is a sufficient statistic for ascertaining burnout and an interval scale transformation is available. The scale as a whole was perfectly targeted to the joint sample. A cut point of 4.4 for severe burnout was chosen at the intersection of the distributions of the clinical and general population.

**Conclusion:**

A revised 18 item version of the SMBQ satisfies modern measurement standards. Using its cut point it offers the opportunity to identify potential clinical cases of burnout.

## Background

Burnout is a mental condition defined as a result of continuous and long-term stress exposure, particularly related to psychosocial factors at work [1]. However, the theoretical basis for the term burnout differs between the available self-report instruments constructed to assess the condition.

The most widely used instrument is the Maslach Burnout Inventory (MBI) and the conceptual basis for MBI is thus often considered as synonymous with the construct burnout. Maslach and colleagues originally defined burnout as a psychological syndrome of emotional exhaustion, depersonalisation (later replaced with the construct cynicism) and reduced effectiveness or personal accomplishment, which makes this scale a multidimensional construct [2,3]. Another conceptual approach was presented by Melamed and co-workers, viewing burnout again as a multidimensional construct consisting of emotional exhaustion, physical fatigue, and cognitive weariness, which together represents the core component of burnout [1,4]. One interesting aspect is that, according to its originator, this latter conceptualization of burnout, although sharing some common variance with depression, represent a separate construct which is not interchangeable with depression [5]. Thus, in clinical populations reporting both burnout, and symptoms of depression and anxiety, it should be possible to follow the course of these conditions separately from each other. Indeed, this conceptualization of burnout has been proven useful, not only to measure burnout in working populations, but also in clinical populations of patients seeking medical care due to stress-related exhaustion [6-8]. In these studies the earlier version of the burnout scale, the Shirom-Melamed Burnout Questionnaire (SMBQ) [9,10] was used, including the subscales," Physical Fatigue", "Cognitive weariness" "Tension", and "Listlessness". Later development of the instrument has resulted in the Shirom-Melamed Burnout Measures (SMBM), which included three subscales; "physical fatigue", "emotional exhaustion" and "cognitive weariness". The burnout construct was not meant to be used in clinical practice as a clinically validated diagnosis. However, it is common for people to seek medical care for severe symptoms of exhaustion related to psychosocial stress, and often these patients fulfill criteria for one or several diagnoses defined under F43 in the International Classification of Diseases (ICD-10) system; Reaction to severe stress, and adjustment disorders. Patients seeking medical care for mental health problems due to long-term stress exposure can report severe symptoms of mental and physical exhaustion and cognitive impairment, all of which are core components of burnout. In this context, the evaluation of the severity of the illness, and/or the measurement of the outcome of treatment, could be undertaken by using an existing burnout questionnaire. The concept of burnout as defined by Shirom and co-workers seems to be suitable for this purpose [2,5], but has been validated and tested mainly in different working populations. Consequently, is potentially useful to ascertain the properties of the SMBQ when used for clinical purposes. The rationale of using the SMBQ rather than the later revised version SMBM is that the latter is explicitly tailored for assessment of working populations [5], including questions of work-related conditions, and relations to co-workers and customers, rather than to patients in clinical settings, some of whom may not be currently working. However, to-date there has been virtually no evidence to support the psychometric attributes of the SMBQ, other than the reported reliability in the original development papers [9,10].

Thus this paper seeks to examine the construct validity of the SMBQ in patients with clinically diagnosed stress-related exhaustion, through both classical and modern test theory approaches, including Confirmatory Factor Analysis (CFA) and Rasch analysis [11].

## Methods

### The shirom-melamed burnout questionnaire

The Shirom-Melamed Burnout Questionnaire (SMBQ) contains 22 items in four subscales: "Physical Fatigue (PF)", "Cognitive weariness (CW)" [9] "Tension", and "Listlessness" [10]. The Physical Fatigue domain consists of 8 items, examples of which are "I feel tired" and "My batteries are dead." Six items measure Cognitive Weariness, examples of which are "I feel I am not thinking clearly" and "I have difficulty thinking about complex things." Four items measure Tension, and include "I feel tensed" and "I feel relaxed". Items measuring Listlessness include "I feel full of vitality" and "I feel alert". Each item is rated using a seven-point scale ranging from 1 'Never or almost never' to 7 'Always or almost always'. Five of the items have reversed scoring, one item in the tension domain, three in the listlessness domain and one in the physical fatigue domain. For each sub-domain, and the scale as a whole, the total score is averaged by dividing by the number of items in the domain.

### Subjects and setting

Data from both a clinical and general population samples were included in the study. The clinical population consisted of patients seeking medical care at a specialized outpatient stress clinic; the Institute of Stress Medicine (ISM) located in Gothenburg, Sweden. All patients were ambulatory at the time of the study and none had received inpatient care due to their illness. They were referred from primary care units or occupational health care centres from the western part of Sweden and the referral criteria were stress-related exhaustion and a maximal duration of sick leave of six months. The patients included in this study were recruited between 2004 and 2009. All patients fulfilled the ICD-10 criteria for "other reaction to severe stress "(F.43.8A), which in Sweden has been further defined with diagnostic criteria of exhaustion which requires the presence of one or several clearly identifiable strain factors during at least six months [12]. During this period 354 patients were referred to the clinic and met these criteria, so entering the treatment program and thus were followed-up. To ensure that the exhaustion experienced by the patients is not due to other known causes, patients with known systemic or psychiatric disease (except depression, anxiety and exhaustion), present infection, body mass index below 18.5 or over 30 kg/m2, vitamin B12 deficiency, thyroid disorder or over-consumption of alcohol were excluded. Pregnant or breast-feeding patients were also excluded

Subjects from the general population were obtained from a survey study with the general aim to investigate different aspects of psychosocial work environment, stress, and stress-related health. This study population comprised a random sample (N = 5,300) of the 48,600 employees of Region Västra Götaland, a provider of public health care, and a random sample (N = 700) of the 2,200 social insurance office workers in the same geographical area. Inclusion criteria of at least one-year duration of employment (at least 50% of full-time) were applied. A postal questionnaire was used and the response rate after two reminders was 61%; thus in total 3,717 subjects responded. The majority was females (87%) and the average age of the participants was 47 years. From this population a stratified age-gender sample, comparable to the patient population, was randomly selected (n = 319). This was to ensure that the full range of burnout (e.g. low to high) was available to the psychometric analysis.

### Internal construct (factorial) validity

#### Factor analysis

The paucity of published evidence concerning the factorial structure of the original SMBQ led to an initial exploration of its structure with a Confirmatory Factor Analysis (CFA) [13]. Both a single unidimensional solution was tested (i.e. all 22 items together) together with a four factor solution, representing the four domains listed above. A robust weighted least squares estimator (WLSMV) for categorical variables was chosen. Fit statistics chosen for this analysis were the Comparative Fit Index (CFI), Tucker-Lewis Index (TLI) and the Root Mean Square Error of Approximation (RMSEA), with guidelines for appropriate fit being > 0.95; > 0.95, and < 0.08 respectively [13]. Modification Indices were examined to give insight into possible structural aspects of model misfit (e.g. local dependency).

An Exploratory Factor Analysis (EFA) was undertaken where the CFA failed, in order to gain further insight into a possible item structure which would be appropriate for the Rasch analysis (a confirmatory procedure). A Promax non-orthogonal rotation method was used, allowing for correlated factors.

#### Rasch analysis

The Rasch model is the formal measurement model required to construct quantitative measurement from dichotomous or ordinal data [11,14,15]. It is used whenever a set of items are intended to be summed together to give a total score. The pattern of responses from such data is checked against the model expectations, which is a parametric probabilistic form of Guttman Scaling [16].

Thus the process of Rasch analysis is concerned with testing to see if the data accord to model expectations, satisfy the various assumptions of the model, and other key measurement issues such as the absence of differential item functioning [17]. For example, the assumption of local independence can be characterised as comprising two elements, response dependency and trait dependency [18]. The former is where items are linked in some way, such as a series of walking items reflecting increasing distances. The latter is multidimensionality. Both these are tested by analysis of the residuals where the former is judged to be absent when residual correlations are below 0.3, and the latter to be unidimensional where patterns of items in the residuals (as identified by a Principal Component Analysis - PCA) are shown to give similar person estimates [19]. Response dependency can be accommodated by grouping locally dependent sets of items into 'testlets' [20]. Where testlets of different lengths are constructed the item residual standard deviation may be inflated.

Another assumption is that of the stochastic ordering of items, testing the probabilistic Guttman pattern. This is confirmed by a series of fit statistics, where Chi-Square based statistics are shown to be non-significant (i.e. no deviation from model expectation) after adjustment for multiple testing [21]. Summary residual statistics, under conditions of perfect fit, are expected to have a mean of zero and standard deviation of one, whereas in practice the latter should be below 1.4, except where testlets have been used to accommodate local dependency issues, when the standard deviation becomes inflated [22]. Individual item residuals are expected to be within the range ± 2.5. Differential Item Functioning (DIF) is deemed absent when there is no significant difference in the residuals (via ANOVA) across key contextual groups, such as age or gender. For analysis of DIF three age groups were used: persons under and up to 38 years (N = 116), 39 to 46 years (N = 99) and persons 46 years or older (N = 104). These groups were based upon distribution to obtain similar numbers within groups to support an ANOVA analysis of the residuals.

Reliability is reported as a Person Separation Index, similar to Cronbach's alpha when data are normally distributed. As both items and persons are calibrated on the same metric, where data fit the Rasch model it is possible to examine the targeting of the items in the scale. A properly targeted instrument would have a mean population value of zero logits, which is also where the items of the scale are centred. Also, when data fit the model, a raw score-interval scale transformation becomes available. This means that the ordinal score, achieved by simply summing the items together, can be transformed into an interval scale latent estimate for use in parametric statistics, and for calculating change scores. This is available because under the Rasch model the raw score is a sufficient statistic for the estimate of the person ability, and the property of specific objectivity (parameter separation) fulfils the requirements to satisfy the axioms of conjoint measurement to provide interval scaling [23-26]. In summary the process of Rasch analysis tests the viability of sets of items to be used as valid and reliable additive scale, including aspects of invariance across groups, and compliance with the requirements for constructing interval scale measurement. Further details of the process are given elsewhere [27-29].

The sample size of 638 is sufficient for both a factor analysis of 22 items, and to give a high degree of precision (i.e. item location estimates within 0.3 logit with 99% confidence) for the Rasch analysis [30].

The study was approved by The Regional Ethical Review Board in Gothenburg and conduced in compliance with the Helsinki declaration. All subjects included in the study signed a written informed consent allowing their data to be used for research purposes.

The Rasch software used was RUMM2030 [31]. CFA and EFA in MPlus6 [32] and all other analysis in SPSS Version 18 [33].

## Results

### Population characteristics

Of the 354 eligible patients, 319 patients (219 women and 100 men) with a mean age of 42 years (SD 9.5) were included in this study, as 35 patients did not consent that their data could be used for research purposes. Sixty-six percent of the patients had a university level education. Profession or type of work was used to categorize educational level in the working population sample as short or long (university level). Sixty-eight patients were working full-time (21%), 63 were on part-time sick leave (20%) and 188 on full-time sick leave (60%). Among the working population sample, 285 individuals reported that they were working either full-time or part-time (89%) while only 12 individuals reported whole or partial sick-leave or sickness benefits at the time of the study (4%). The remaining reported other types of leave, mainly parental leave. Thus in the pooled data 416 (65%) persons were working full or part time.

### Internal construct and factorial validity

A Confirmatory Factor Analysis (CFA) of a single overall score from all 22 items was unsupported (RMSEA 0.170; CFI 0.946; TLI 0.940). However, modification indices suggested extensive correlation of errors, mostly within the four underlying domains. Also a CFA did not support the original published four factor structure (RMSEA 0.133; CFI 0.977; TLI 0.974).

In contrast an Exploratory Factor Analysis (EFA) partially supported the four factor solution (RMSEA 0.056; RMSR 0.012), but with significant cross-loadings between the 'burnout' and 'listlessness' domains. The tension and mental tiredness domains were fully supported. Given the failure of the CFA, and the suggestion by the Modification Indices that the errors of many items should be correlated, this suggests that local dependency among clusters of items may have contributed to the failure, and that these are predominately found within the items sets of the four domains.

When the data was fitted to the Rasch model, this was confirmed. The Rasch analysis showed that most items had ordered thresholds, with only minimal disordering of categories in two items. However, within the 22 item set, the analysis identified a considerable breach of the assumption of local independence, with correlated residuals clustering within the four domains, with consequent lack of fit to the model (Table 1, Analysis 1). Consequently, the items from within each domain were grouped as a testlets. With four such testlets, the data failed to fit model expectations with the 'tension' testlet showing considerable misfit. (Analysis 2). After removal of the tension testlet, fit of the data to the model was good (Analysis 3).

**Table 1:** Fit to the Rasch model

	Item Fit Residual		Person Fit Residual		Chi Square Interaction			PSI	Unidimensionality	
**Analysis**	**Mean**	**SD**	**Mean**	**SD**	**Value**	**df**	**p**		**% Sig**	**% at LCI**

1, 22 items	0.741	4.005	-0.326	1.792	562.4	198	< 0.001	0.96669	10.10%	8.40%

2, 4 testlets	-0.256	3.692	-0.463	1.075	56.77	36	0.01515	0.92432	3.20%	0.01%

3, 3 testlets	-0.016	2.689	-0.444	0.962	32.28	27	0.22175	0.91544	3.25%	1.50%

4, Tension	-0.315	1.978	-0.5	0.958	33.18	27	0.19132	0.86666	1.90%	0.01%

**Figure 1:**
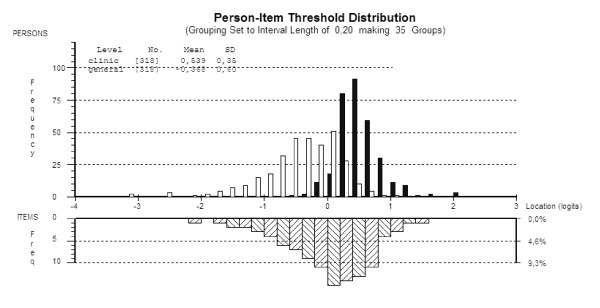
**Distribution of General Population of Health Care Workers (white) and Clinical (Black) subjects and items on the same metric**.

No differential item functioning was observed for age, gender, or sample. In the latter case this shows that the (revised 18 item) scale is invariant across the general population and a clinical sample. The revised scale was strictly unidimensional in that estimates taken from different subsets of items, identified by those loading positive and negative on the first residual component of the residuals, showed no significant difference. Some reduction in reliability was observed as a consequence of accommodating the local dependency through the testlet design, but the level of reliability remained consistent with individual clinical use. Figure 1 shows how the total score (omitting the tension items) is distributed across the general and clinical populations. As expected there is a significant difference between the two groups (F:537.9; p = < 0.001).

Fit of the remaining Tension domain achieved fit to the model after removing the item "I feel restless" (Analysis 4).

**Figure 2:**
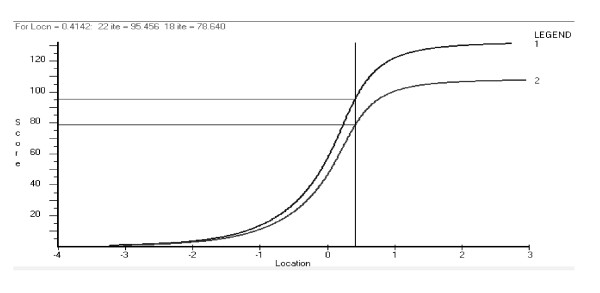
**Equating of scores for the 22 item SMBQ version (1) with the 18 item SMBQ version (2)**.

In the 18 item revised version (based upon three testlets) a cut point for severe burnout was chosen at the intersection of the distributions of the clinical and general population. This equated to a value of 4.4 on the revised 18 item scale (raw score of 79/18), and 4.4 on the original 22 item scale (raw score of 96/22) (Figure 2). In the current study this would place 83.4% of the clinical sample above the cut, and 86.5% of the general population sample of health care and social insurance workers below the cut.

A raw score - interval scale conversion is available for the revised (18 item scale) scale, to facilitate use in outcome studies and other situations when the calculation of change scores is required (Table 2). This conversion is based upon the testlet design such that the transformed score is adjusted for local dependency and consequently unbiased.

**Table 2:** Raw-score metric conversion of the 18 -item revised scale

Average Value	Raw Score	Transformed Score	Average Value	Raw Score	Transformed Score	Average Value	Raw Score	Transformed Score
1.0	18	18.00	2.2	39	65.41	3.3	60	74.87

1.1	19	29.67	2.2	40	66.03	3.4	61	75.22

1.1	20	37.00	2.3	41	66.61	3.4	62	75.55

1.2	21	41.57	2.3	42	67.16	3.5	63	75.87

1.2	22	44.90	2.4	43	67.70	3.6	64	76.20

1.3	23	47.51	2.4	44	68.22	3.6	65	76.53

1.3	24	49.65	2.5	45	68.74	3.7	66	76.83

1.4	25	51.47	2.6	46	69.22	3.7	67	77.14

1.4	26	53.09	2.6	47	69.70	3.8	68	77.43

1.5	27	54.53	2.7	48	70.16	3.8	69	77.74

1.6	28	55.82	2.7	49	70.61	3.9	70	78.03

1.6	29	57.03	2.8	50	71.05	3.9	71	78.32

1.7	30	58.13	2.8	51	71.47	4.0	72	78.60

1.7	31	59.15	2.9	52	71.87	4.1	73	78.89

1.8	32	60.11	2.9	53	72.28	4.1	74	79.18

1.8	33	61.01	3.0	54	72.68	4.2	75	79.45

1.9	34	61.86	3.1	55	73.07	4.2	76	79.74

1.9	35	62.65	3.1	56	73.45	4.3	77	80.01

2.0	36	63.39	3.2	57	73.82	4.3	78	80.28

2.1	37	64.09	3.2	58	74.18	4.4	79	80.56

2.1	38	64.76	3.3	59	74.53	4.4	80	80.83

Average Value	Raw Score	Transformed Score	Average Value	Raw Score	Transformed Score	Average Value	Raw Score	Transformed Score

4.5	81	81.10	5.7	102	87.33	6.8	123	105.00

4.6	82	81.39	5.7	103	87.70	6.9	124	108.79

4.6	83	81.64	5.8	104	88.06	6.9	125	115.19

4.7	84	81.93	5.8	105	88.45	7.0	126	126.00

4.7	85	82.20	5.9	106	88.85			

4.8	86	82.47	5.9	107	89.27			

4.8	87	82.76	6.0	108	89.70			

4.9	88	83.03	6.1	109	90.16			

4.9	89	83.31	6.1	110	90.66			

5.0	90	83.58	6.2	111	91.18			

5.1	91	83.87	6.2	112	91.74			

5.1	92	84.16	6.3	113	92.33			

5.2	93	84.45	6.3	114	92.99			

5.2	94	84.76	6.4	115	93.70			

5.3	95	85.06	6.4	116	94.49			

5.3	96	85.37	6.5	117	95.35			

5.4	97	85.68	6.6	118	96.33			

5.4	98	85.99	6.6	119	97.47			

5.5	99	86.31	6.7	120	98.79			

5.6	100	86.64	6.7	121	100.37			

5.6	101	86.99	6.8	122	102.37			

## Discussion

Data from a clinical and working population samples showed that the Shirom-Melamed Burnout Questionnaire (SMBQ) satisfies Rasch model expectations after the removal of 4 of the original items measuring tension. There was some local dependency in the data, marginally inflating reliability, but not sufficiently to compromise the use of the scale in a clinical setting. The total raw score from the revised 18 item scale score is a sufficient statistic for ascertaining burnout, according to the definition associated with this instrument, and an interval scale transformation is available. The scale as a whole was perfectly targeted to the joint sample.

Both the CFA and Rasch analysis identified that there were problems with the dimensionality of the original 22 item scale, and both indicated that the tension set of items were the problem, as well as local dependency throughout the scale.

Consequently the revised scale omits the 'tension' set of items, which is consistent with the later SMBM scale, which also omitted these items. Thus the revised scale operationalises burnout by the sub-domains of listlessness, physical fatigue and cognitive weariness. This would still differ somewhat from the SMBM which also includes the subscales physical fatigue and cognitive weariness, but instead of the subscale listlessness, emotional exhaustion is included, containing three items that are all related to contact with co-workers and customers.

Given we were searching for a scale useful to measure burnout in clinical settings, where many individuals were not currently working, the forerunner of SMBM was considered more meaningful and suitable for this purpose. Likewise, when initially choosing between different burnout instruments, the most frequently used burnout instrument, the Maslach burnout inventory (MBI) was not chosen, primarily as this scale contains three multidimensional subscales; exhaustion, cynicism, and reduced personal efficacy, all of which are strongly related to the current situation at work.

One important aspect when measuring symptoms of burnout and exhaustion in a clinical population is the possibility to follow the course of symptoms over time. This could be particularly important in the evaluation of the effects of treatment and rehabilitation of patients suffering from stress-related exhaustion or burnout. Co-morbid depression is common in patients seeking medical care for symptoms of burnout and exhaustion [8,9], but it has been suggested from previous research that burnout and depression are two distinct constructs [1]. In our clinical experience some of these patients have a history of depression, and thus vulnerability for developing this co-morbidity when exposed to prolonged, high stress exposure, while others develop depressive symptoms as a response to the exhaustion and cognitive impairment. Consequently, we propose that this operationalised burnout construct can be used to separate symptoms of burnout and depression in a clinical population, but this needs to be confirmed in future studies.

There are a number of limitations to this study. The population sampled may be considered to be at the extremes of the continuum of the latent construct of burnout - i.e. none/mild and extreme. However, the distribution between the pooled sample was overlapping and, furthermore, the Rasch estimates of item difficulty are independent of the distribution of the sample of persons used for the calibration, courtesy of the property of specific objectivity which is unique to the Rasch model [11]. The population study is also solely health and social care workers, and other occupational groups will need to be sampled in the future.

As it has been suggested that both work-related and family-related stress exposure contributes to exhaustion in both clinical and non-clinical populations [7,10], future research will also offer the opportunity to explore if burnout and depression are differently related to work-related stress exposure compared to domestic related stress-exposure. Further work might also include comparison of different scales in the same populations to help understand in what way, if at all, they differ. The role of burnout screening questionnaires in proactive programs to prevent onset of severe burnout also needs to be considered.

Finally, a better understanding of the place of burnout within the broader psychosocial model, including potential moderators and mediators should offer considerable potential for future research activity.

## Conclusion

In conclusion, a shorter 18 item version of the Shirom-Melamed Burnout Questionnaire (SMBQ-18) satisfies current standards of measurement, and provides a potential useful screening tool and outcome measure for use in clinical settings where patients may not have been in work for some time.

## Competing interests

The authors declare that they have no competing interests.

## Authors' contributions

The authors contributed equally to this work. All authors read and approved the final manuscript.

## Pre-publication history

The pre-publication history for this paper can be accessed here:

http://www.biomedcentral.com/1471-2458/12/1/prepub

## References

[B1] Melamed S Shirom A Toker S Berliner Shapira S Burnout and risk of cardiovascular disease: evidence, possible causal paths, and promising research directions Psychol Bull 2006 132 3 327 353 16719565 10.1037/0033-2909.132.3.327

[B2] Shirom A Melamed S A comparison of the construct validity of two Burnout measures in two groups of professionals Int J Stress Manag 2006 13 2 176 200

[B3] Maslach C Schaufeli WB Leiter MP Job Burnout Annu Rev Psychol 2001 52 397 422 10.1146/annurev.psych.52.1.397 11148311

[B4] Shirom A Cooper CL, Robertson I Burnout in work organization International Review of Industrial and Organizational Psychology 1989 New York: Wiley

[B5] Shirom A Quick C, Tetrick LE Job-related burnout: A review Handbook of occupational health psychology 2003 Washington, DC 245 265 American Psychological Association

[B6] Grossi G Perski A Ekstedt M Johansson T Lindstrom M Holm K The morning salivary cortisol response in burnout J Psychosom Res 2005 59 2 103 111 10.1016/j.jpsychores.2005.02.009 16186006

[B7] Stenlund T Ahlgren C Lindahl B Burell G Knutsson A Stegmayr B Birgander LS Patients with burnout in relation to gender and a general population Scand J Public Health 2007 35 5 516 523 10.1080/14034940701271874 17852977

[B8] Jonsdottir IH Hagg DA Glise K Ekman R Monocyte chemotactic protein-1 (MCP-1) and growth factors called into question as markers of prolonged psychosocial stress PLoS One 2009 4 11 e7659 10.1371/journal.pone.0007659 19888340 PMC2766003

[B9] Melamed S Kushnir T Shirom A Burnout and risk factors for cardiovascular diseases Behav Med 1992 18 53 60 10.1080/08964289.1992.9935172 1392214

[B10] Kushnir T Melamed S The Gulf War and its impact on burnout and well-being of working civilians Psychol Med 1992 22 987 95 10.1017/S0033291700038551 1488493

[B11] Rasch G Probabilistic models for some intelligence and attainment tests 1960 Chicago: University of Chicago Press

[B12] National Board of Health and Welfare Exhaustion disorder (Swedish: Utmattningssyndrom-stressrelaterad psykisk ohälsa) Stockholm 2003

[B13] Brown TA Confirmatory Factor Analysis: for Applied Research 2006 New York: The Guilford Press

[B14] Andrich D Rating formulation for ordered response categories Psychometrika 1978 43 561 573 10.1007/BF02293814

[B15] Masters G A Rasch model for partial credit scoring Psychometrika 1982 47 149 174 10.1007/BF02296272

[B16] Guttman LA Stouffer SA, Guttman LA, Suchman FA, Lazarsfeld PF, Star SA, Clausen JA The basis for Scalogram analysis Studies in social psychology in World War II: Vol 4. Measurement and Prediction 1950 Princeton: Princeton University Press 60 90

[B17] Teresi JA Kleinman M Ocepek-Welikson K Modern psychometric methods for detection of differential item functioning: application to cognitive assessment measures Stat Med 2000 19 1651 83 10.1002/(SICI)1097-0258(20000615/30)19:11/12<1651::AID-SIM453>3.0.CO;2-H 10844726

[B18] Marais I Andrich D Formalizing Dimension and Response Violations of Local Independence in the Unidimensional Rasch Model J Appl Meas 2008 9 3 200 15 18753691

[B19] Smith EV Detecting and evaluation the impact of multidimensionality using item fit statistics and principal component analysis of residuals J Appl Meas 2002 3 205 231 12011501

[B20] Wainer H Kiely G Item clusters and computer adaptive testing: A case for testlets J Educ meas 1987 24 185 202 10.1111/j.1745-3984.1987.tb00274.x

[B21] Bland JM Altman DG Multiple significance tests: the Bonferroni method Brit Med J 1995 310 170 10.1136/bmj.310.6973.170 7833759 PMC2548561

[B22] Steinberg L Thissen D Uses of Item Response Theory and the Testlet Concept in the Measurement of Psychopathology Psychol Methods 1996 1 81 97

[B23] Luce RD Tukey JW Simultaneous conjoint measurement: A new type of fundamental measurement J Math Psychol 1964 1 1 27 10.1016/0022-2496(64)90015-X

[B24] Perline R Wright BD Wainer H The Rasch model as additive conjoint measurement Appl Psycho Meas 1997 3 237 256

[B25] Karabatos G The Rasch model, additive conjoint measurement, and new models of probabilistic measurement theory J Appl Meas 2001 2 389 423 12011506

[B26] Van Newby A Conner GR Bunderson CV The Rasch model and additive conjoint measurement J Appl Meas 2009 10 348 354 19934524

[B27] Pallant JF Tennant A An introduction to the Rasch measurement model: An example using the Hospital Anxiety and Depression Scale (HADS) Brit J Clin Psych 2007 46 1 18 10.1348/014466506X96931 17472198

[B28] Tennant A Conaghan PG The Rasch measurement model in rheumatology: What is it and why use it? When should it be applied, and what should one look for in a Rasch paper? Arthritis Rheum 2007 57 1358 1362 10.1002/art.23108 18050173

[B29] Hagquist C Bruce M Gustavsson JP Using the Rasch model in nursing research: an introduction and illustrative example Int J Nurs Stud 2009 46 3 380 93 10.1016/j.ijnurstu.2008.10.007 19059593

[B30] Linacre JM Sample size and item calibration stability RMT 1994 7 4 328 31

[B31] Andrich D Lyne A Sheridan B Luo G RUMM 2030 2010 Perth: RUMM Laboratory

[B32] Muthen LK Muthen BO Mplus User's Guide 2010 Sixth Los Angeles CA: Muthen & Muthen

[B33] SPSS Inc 2009

